# Core Fucosylation of Intestinal Epithelial Cells Protects Against *Salmonella* Typhi Infection via Up-Regulating the Biological Antagonism of Intestinal Microbiota

**DOI:** 10.3389/fmicb.2020.01097

**Published:** 2020-05-27

**Authors:** Sijia Hao, Qingjie Fan, Yaqiang Bai, Hui Fang, Jiaorui Zhou, Tomohiko Fukuda, Jianguo Gu, Ming Li, Wenzhe Li

**Affiliations:** ^1^College of Basic Medical Sciences, Dalian Medical University, Dalian, China; ^2^Institute of Molecular Biomembrane and Glycobiology, Tohoku Medical and Pharmaceutical University, Sendai, Japan

**Keywords:** core fucosylation, gut microbiota, *S.* Typhi infection, Wnt signaling pathway, *Lactobacillus*

## Abstract

The fucosylated carbohydrate moieties on intestinal epithelial cells (IECs) are involved in the creation of an environmental niche for commensal and pathogenic bacteria. Core fucosylation catalyzed by fucosyltransferase 8 (Fut8) is the major fucosylation pattern on the *N*-glycans of the surface glycoproteins on IECs, however, the role of IECs core fucosylation during infection remains unclear. This study was conducted to investigate the interaction between IECs core fucosylation and gut microbiota, and the effects of this interaction on protecting *Salmonella enterica* subsp. *enterica* serovar Typhi (*S.* Typhi) infection. Firstly, the *Fut8*^+/+^ and *Fut8*^+/–^ mice were infected with *S.* Typhi. The level of IECs core fucosylation and protein expression of intestinal mucosa were then detected by LCA blot and Western blot, respectively. The gut microbiota of *Fut8*^+/+^ and *Fut8*^+/–^ mice before and after *S.* Typhi infection was assessed by 16S rRNA sequencing. Our results showed that core fucosylation was ubiquitous expressed on the intestinal mucosa of mice and had significant effects on their gut microbiota. Fut8^+/–^ mice was more susceptive to *S.* Typhi infection than Fut8^+/+^ mice. Interestingly, infection of *S.* Typhi upregulated the core fucosylation level of IECs and increased the abundances of beneficial microorganisms such as *Lactobacillus* and *Akkermansia* spp. Further *in vitro* and *in vivo* studies demonstrated that Wnt/β-catenin signaling pathway mediated the elevation of IECs core fucosylation level upon infection of *S.* Typhi. Taken together, our data in this study revealed that the IECs core fucosylation plays an important role in protecting against *S.* Typhi infection via up-regulating the biological antagonism of intestinal microbiota.

## Introduction

Intestinal mucosa creates a basic and physical barrier between the gut microbes and the host tissues. The fucosylation of intestinal epithelial cells (IECs) is closely related to the immune function of intestinal mucosa, and can prevent against pathogen infection, and the development of colitis and colon cancer (Goto et al., 2016; Yu et al., 2019). The core fucosylation catalyzed by fucosyltransferase 8 (*Fut8*) is the major fucosylation pattern on the *N*-glycans of the surface glycoproteins on IECs. Fut8 transfers the guanosine diphosphate-fucose (GDP-Fucose) to the sixth carbon atom on the *N*-acetylglucosamine (GlcNAc) of the *N*-glycan, forming a α-1, 6-glycosidic bond which is called as core fucosylation (Miyoshi et al., 1999; Calderon et al., 2016). Although many studies support that *Fut8* gene knockout has an great impact on the function of glycoproteins which is exerting an enormous function on immune responses, such as cell recognition and information transferation, no study has investigated the role of IECs core fucosylation during infection (Liang et al., 2018).

Intestinal commensal bacteria play an important role in immune system. IECs send signals which are constituted by glycosylation, protein decoration and other signals to mucosal immune cells to make an interaction with commensal bacteria and IECs to establishing intestinal immunological homeostasis (Goto and Kiyono, 2012). The glycosylation of IECs contributes to the resistance to intestinal pathogens, and has a significant impact on shaping commensal bacteria (Hooper et al., 1999). Studies have shown that commensal bacteria are able to utilize fucoses, especially *Bacteroides* spp., as components of the bacterial outer membrane, or using them as a nutrient (Leis et al., 1997; Coyne et al., 2005). However, the interaction between IECs core fucosylation and gut microbiota, and whether this interaction affects immune responses during infection remain unclear.

The infection of pathogenic *Salmonella* spp. is a major public health concern all around the world. It’s chronic colonization in human intestine is also a risk factor for the development of colorectal cancer (CRC) and inflammatory bowel disease (IBD) (Gradel et al., 2009; Kato et al., 2013). *Salmonella enterica* serotype typhi mainly cause typhoid fever, a systemic infection, at least 16 million new cases and 600,000 deaths each year (Maskalyk, 2003). Changes of gut microbiota and signaling pathways of IECs were both found involved in *Salmonella* spp. infection (Liu et al., 2010; Wang et al., 2018). Among them, the Wnt signaling pathway was found to be involved in *Salmonella* infection, enhanced accumulation of nuclear β-catenin and in turn activated the transcription of its target genes (Lawhon et al., 2011; Kogut and Arsenault, 2015). Wnt signaling pathway was also found to be correlated with the hyper-expression of core fucosylation in breast cancer through activation of the *Fut8* gene transcription (Yang et al., 2017). It was also known to play an important role in the development and differentiation of immune cells, and regulate pathogen-induced inflammation and mucosal tolerance by antigen presenting cells (APCs) (Staal et al., 2008; Maloy and Powrie, 2011; Rescigno, 2011; Swafford et al., 2018). We therefore asked that whether *Salmonella* infection induce alteration of IECs core fucosylation through activating Wnt signaling pathway, and whether this alteration can affect gut homeostasis.

In this study, we provide the first confirmation that the deficiency of core fucosylation in mice resulted in severe infection when challenged by *Salmonella enterica* subsp. *enterica* serovar Typhi (*S.* Typhi), and this was correlated with changing of gut microbiota and activated Wnt signaling pathway in IECs. The *S.* Typhi-induced activation of Wnt signaling pathway contributed to the rapid elevation of IECs core fucosylation, which in turn promoted the dominant growth of fucose-utilizing bacteria such as *Lactobacillus* and *Akkermansia*, to resist the invasion of *S.* Typhi. Our study thus suggests that Wnt signaling pathway is involved in the regulation of IECs core fucosylation, which contributes to the protection against *S.* Typhi infection via up-regulating the biological antagonism of intestinal microbiota.

## Materials and Methods

### Mice

*Fut8*^+/–^ mice were generated according to the methods from the studies of Wang et al. (2005) and Li et al. (2006). Wild type *Fut8*^+/+^ and heterozygous *Fut8*^+/–^ mice with the Institute of Cancer Research (ICR) background were maintained in a room illuminated for 12 hours (h) (08:00–20:00) and kept at 24 ± 1°C with free access to food and water in the specific pathogen-free laboratory animal facility of Dalian Medical University, Dalian, China. Animal experiments of *S.* Typhi-infected mouse model were performed by using specific pathogen-free ICR mice that were 6–7 weeks old. All *Fut8*^+/+^ and *Fut8*^+/–^ mice were obtained by crossing *Fut8*^+/–^ female and male mice, and they were nurtured by *Fut8*^+/–^ maternal mice to exclude the influence of maternal milk glycans on gut microbiota of the neonates. Due to the low body weight, low birth rate and high death rate of *Fut8*^–/^*^–^* mice, we adopted the *Fut8*^+/–^ mice in this study to establish to the infection models as the growth of these mice are comparable with *Fut8*^+/+^ mice.

Mice were infected with the indicated *S.* Typhi strain by oral gavage at a concentration of 1 × 10^8^ colony-forming units (CFU)/ml, 0.1 ml once a day, for 7 days, and were weighted at 9:00 am everyday. Mice were sacrificed on the morning of day 8 for intestinal tissue, intestinal mucosal and intestinal content collection. Tissue sections were prepared for hematoxylin and eosin (H&E) and immunohistochemical staining. All the animal experiments were conducted according to the National Institutes of Health Guide for the Care and Use of Laboratory Animals (NIH Publication No. 8023).

### Antibodies

Anti-Frizzled 7 (ab64636), anti-β-catenin (ab32572), and FITC-labeled goat anti-rabbit IgG (ab6717) were obtained from Abcam; anti-GAPDH (BP009232MO) were obtained from Cusabio; biotinylated lens culinaris agglutinin (LCA) (Z1010) and fluorescein labeled LCA (FL1041) were purchased from Vector; horseradish peroxidase (HRP)-labeled goat anti-mouse IgG (A0218), HRP-labeled goat anti-rabbit IgG (A0208) and HRP-labeled Streptavidin (A0303) were purchased from Beyotime. Andy Fluor 647 streptavidin secondary antibody (SA2K1469A) was purchased from GeneCopoeia.

### Fecal DNA Extraction, PCR, and 16S rRNA Amplicon Data Processing

Microbial genome DNA was extracted from fecal samples of mice using E.Z.N.A.^®^ Stool DNA kit (Omega Bio-tek, Inc.) according to the manufacturer’s instructions. A Nanodrop 2000 spectrophotometer was used to evaluate the purity and concentration of isolated DNA. Universal primers 338F (5′-ACTCCTACGGGAGGCAGCA-3′) and 518R (5′- ATTACCGCGGCTGCTGG -3′) were used to amplify the V3 hypervariable region of the 16S rRNA from metagenomic DNA in mice feces. The 50-μl PCR mixture contained the following components: 5 μl DNA template, 25 μl PCR 2 × Easy Taq Super mix (HotStarTaq Plus DNA polymerase, dNTPs, MgCl_2_ and reaction buffer), 1 μl of each primer and 18 μl deionized water. The PCR program consisted of an initial step at 95°C for 5 min; 30 cycles of 94°C for 45 s, 55°C for 45 s, and 72°C for 60 s; and a final extension at 72°C for 8 min. PCR amplicons were sequenced and the data were analyzed by Illumina MiSeq (Novogene Bioinformatics Technology Co., Ltd., Beijing, China) (Li et al., 2017). The sequencing data were deposited in NCBI SRA under the accession number PRJNA625618.

### Bacterial Strains and Growth Condition

The *S.* Typhi strain used in this study is *Salmonella enterica* subsp. *enterica* serovar Typhi CMCC (B) 50071 CICC 10871 strain. The *S.* Typhi strain was provided by professor Yongliang Yang at Dalian University of Technology, China. The culture was prepared by inoculating 10 ml of Luria–Bertani broth with 10 μl of a stationary-phase culture followed by overnight incubation (∼18 h) at 37°C.

### Detection of Intestinal sIgA by ELISA

The content of the mouse small intestine was rinsed with PBS (5 ml) and centrifuged at 10,000 rpm for 10 min, and the supernatant was collected for sIgA detection by ELISA kit (Shanghai Langton Biological Technology Co., Ltd., China) according to the manufacturer’s instructions. The absorbance was measured at 450 nm using a computer-interfaced microplate reader (Bio-Rad, Houston, TX, United States).

### Western Blot and Lectin Blot Analysis

Cells were washed with phosphate buffer solution (PBS) and then lysed with ice-cold buffer (50 mM Tris-HCl, 150 mM of NaCl, 1% Triton X-100, 2 mM EDTA, supplemented with 0.1 mM phenylmethylsulfonyl fluoride). The intestinal mucosa of mice was lysed with ice-cold buffer (50 mM Tris-HCl, 150 mM of NaCl, 1% Triton X-100, 2 mM EDTA, supplemented with 0.1 mM phenylmethylsulfonyl fluoride). Total protein concentrations were determined by bicinchoninic acid protein assay (BCA Protein Assay Kit, Pierce, Rockford, IL, United States). 10 μl of proteins was subjected to SDS-PAGE. After SDS-PAGE, the proteins were transferred to polyvinylidene difluoride (PVDF) membranes immunoblot or lectin blot. Following incubation with the appropriate primary antibodies first antibody anti-Frizzled 7, anti-β-catenin, anti-GAPDH or the biotinylated LCA, which preferentially recognizes the core fucose, followed by incubation with HRP-conjugated secondary antibody or HRP-conjugated streptavidin for 1h at room temperature. After washing, the membranes were visualized by chemiluminescence using an ECL kit (Pierce, Rockford, IL, United States).

### Immunofluorescence

Formalin-fixed ovarian tissue specimens were paraffin-embedded. For immunohistochemical analysis, the specimens were deparaffinized twice in xylene and hydrated through a graded series of ethanol to PBS. The endogenous peroxidase activity was blocked with 3% H_2_O_2_ for 10 min and then in 5% BSA in PBS for 30 min to reduce non-specific background. The specimens were incubated with biotinylated LCA (1:600) or anti-β-catenin (1: 200) for 10 to 12 h at 4°C. Then the specimens were incubated with Andy Fluor 647 streptavidin and FITC-labeled goat anti-rabbit IgG secondary antibody for 1 h at 37°C, and incubated with DAPI (C0060, Solarbio) for 10 min at room temperature. Other specimens were directly incubated with fluorescein labeled LCA for 10 to 12 h at 4°C. Finally, the slides were examined under a fluorescence microscope (Leica, United Kingdom) at a 200× magnification using the appreciate filter. The specimens were analyzed by hematoxylin-eosin (H&E) staining.

### Histopathological Evaluation

The specimens were strained with hematoxylin-eosin (H&E) and pathology was quantified as previously described, evaluating submucosal edema, PMN infiltration, goblet cells and epithelial damage yielding a total score of 0–13 points (Hapfelmeier et al., 2008).

### Cells and Culture Conditions

Caco-2 cells were maintained in Dulbecco’s modified Eagle’s medium (DMEM) supplemented with 10% fetal bovine serum (FBS), and maintained at 37°C with 5% CO_2_ as previously described (Liu et al., 2011).

Cells were inoculated in 6-well plates for 24 h and then replaced. The cells were divided into control group (group 0) (10^6^ cells) and experimental group (group 10, 100, and 200 ng) (10^6^ cells) according to the completely random method. The group 10 ng was given 10^3^ ng/mL Dkk-1 (HY-P7155A, MCE) 10 μl, the group 100 ng was given 10^4^ ng/mL Dkk-1 10 μl, the group 200 ng was given 2 × 10^4^ ng/mL Dkk-1 10 μl, and the same volume PBS was added into group 0. Four groups of cells were taken after 48 h of intervention. After rinsing with PBS and centrifugation at 1,200 rpm/min for 3 min, cell lysis was added. The total protein was extracted by lysis on ice for 30 min.

### *S.* Typhi Invasion Assay

To determine the effect of *S.* Typhi infection to Caco-2 cells, Caco-2 cells were incubated with the *S.* Typhi strain (∼20 CFU/cell) for 30 min, washed, and incubated in fresh DMEM with 10% FBS for 30, 60, or 120 min (Wang et al., 2018). Control group indicates without bacterial treatment. After rinsing with PBS and centrifugation at 1,200 rpm/min for 3 min, cell lysis was added. The total protein was extracted by lysis on ice for 30 min.

Caco-2 cells were classified into Control group (10^6^ cells without any treatment), Dkk-1 group (10^6^ cells were treated by 2 × 10^4^ ng/mL, 10 μl Dkk-1 for 48 h), *S.* Typhi group (10^6^ cell were incubated with *S.* Typhi strain for 30 min, and incubated with medium for 60 min), Sal + Dkk-1 group (10^6^ cell were incubated with *S.* Typhi strain for 60 min, and incubated with medium for 60 min then treated by 2 × 10^4^ ng/mL, 10 μl Dkk-1 for 48 h).

### Chromatin Immunoprecipitation-qPCR (ChIP-qPCR) Assay

Chromatin immunoprecipitation (ChIP) was performed by using the ChIP Assay Kit (Beyotime Biotechnology) and anti-β-catenin antibody, as previously described (Bernt et al., 2011). Caco-2 cells were classified into Control group (13.7 × 10^6^ cells without any treatment) and *S.* Typhi group (13.7 × 10^6^ cells were incubated with *S.* Typhi strain for 30 min, and incubated with medium for 60 min), crosslinked with 1% formalin, then lysed in SDS buffer and sonication was used to fragment the DNA. ChIP for TCF was performed using an anti-β-catenin antibody. Eluted DNA fragments were analyzed by qPCR. To amplify the human *Fut8* promoter region (−1416 bp to −1409 bp) containing the TCF-binding site, the following primer sets were used: *Fut8* ChIP forward, 5′-CACCCCTTCTTGCTCTTGGC-3′ and *Fut8* ChIP reverse, 5′-GACTGTCAGCCATGGAAGCAT-3′.

### Statistical Analysis

Each experiment was performed at least three times and the mean was used to calculate significance. Data was expressed as mean values ± standard error mean (SEM). Data analysis was performed with the statistical software package Graph Pad Prism 5. Student’s *t*-test was used for statistical analysis between two groups, and the quantitative multiple group comparisons was performed using one-way ANOVA followed by Tukey’s test (compare all pairs of columns). Differences were considered to be statistically significant at ^∗^*p* < 0.05, ^∗∗^*p* < 0.01, ^∗∗∗^*p* < 0.001.

## Results

### The Ubiquitous Core Fucosylation of IECs Affects Intestinal Microbiota in Mouse

**FIGURE 1:**
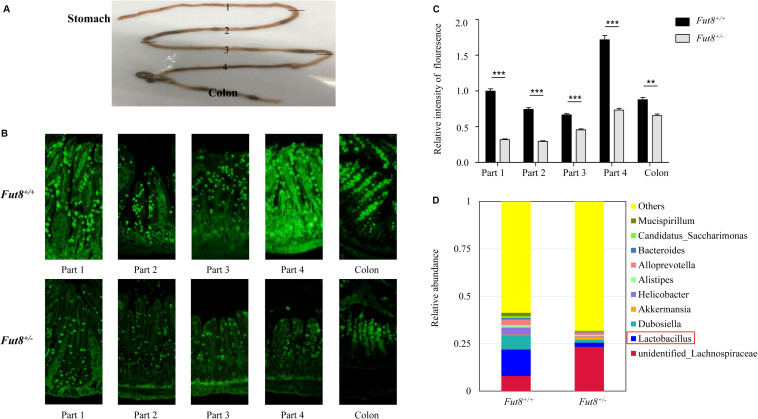
The ubiquitous core fucosylation of IECs affects intestinal microbiota in mouse. **(A)** Mouse intestine was divided into the small intestine and colon parts. And the small intestine was further divided into four parts (parts 1, 2, 3, and 4) equally, from the proximal (duodenum) to the distal (ileum) ends. **(B)** The core fucosylation expression and localization of wild type *Fut8*^+/+^ and heterozygous *Fut8*^+/–^ mice in intestinal mucosa was detected by fluorescein labeled LCA. **(C)** The relative intensity of fluorescence was detected by ImageJ. The core fucosylation level was found higher in part 1, part 4 of small intestine and in the colonic tissues compared to other parts. And there was a significant decrease of the core fucosylation expression of *Fut8*^+/–^ mice compared to *Fut8*^+/+^ (WT) mice. Data are shown as mean values ± SEM (*n* = 3; **p < 0.01; ***p < 0.001). **(D)** The relative abundances of fecal bacterial genera of *Fut8*^+/–^ and *Fut8*^+/–^ mice.

To assess the level of core fucosylation throughout mouse intestine, we first divided the small intestine into four parts (part 1, 2, 3, and 4) equally, from the proximal (duodenum) to the distal (ileum) ends (Figure 1A), and investigated all the 4 parts and the colon by an immunofluorescence assay. Our result showed that core fucosylation was ubiquitous in mouse intestine, especially in colon and the part 1 and part 4 of small intestine (Figures 1B,C). Compared to *Fut8*^+/+^ mice, a significant decrease of the core fucosylation was detected in the intestine of *Fut8*^+/–^ mice.

To examine the effects of core fucosylation on gut microbiota, we compared the microbial structure of *Fut8*^+/+^ and *Fut8*^+/–^ mice by 16S rRNA sequencing. The major bacterial genera in gut of *Fut8*^+/+^ and *Fut8*^+/–^ mice are shown in Figure 1D. Several genera, including the unidentified *Lachnospiraceae* (7.92%), *Lactobacillus* (13.99%), *Dubosiella* (7.56%), and *Akkermansia* (0.30%) were found dominant in gut of *Fut8*^+/+^ mice. In contrast, the *Fut8*^+/–^ mice exhibited an altered gut microbial structure. Compared with *Fut8*^+/+^ mice, a significant increase in the proportion of the unidentified *Lachnospiraceae* (23.37%) was detected in gut of *Fut8*^+/–^ mice, which also harbors remarkably reduced abundance of *Lactobacillus* spp. (2.17%) (Figure 1D). Our data suggested that the core fucosylation of IECs in mice have a strong effect on their gut microbiota.

### *Fut8*^+/–^ Mice Is Susceptive to *S.* Typhi Infection

**FIGURE 2:**
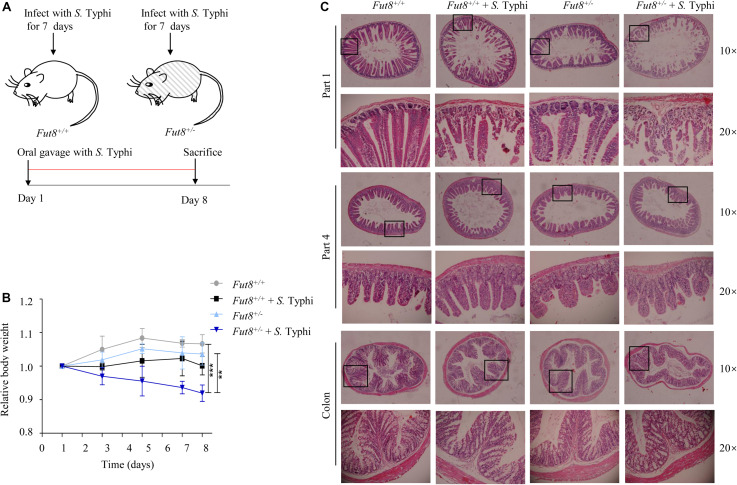
*Fut8*^+/–^ mice is susceptive to *S.* Typhi infection. **(A)** *Fut8*^+/–^ and *Fut8*^+/–^ mice were infected with *S.* Typhi every day by oral gavage for 7 days at a concentration of 1 × 10^8^ CFU/ml. **(B)** Following oral infection with *S.* Typhi, mice were weighed daily and the percent change in weight from days 1 to 8 is shown. Data were analyzed with a one-way ANOVA using Bonferroni correction (*Fut8*^+/+^, *n* = 3; *Fut8*^+/+^ + *S.* Typhi, *n* = 3; *Fut8*^+/–^, *n* = 3; *Fut8*^+/–^ + *S.* Typhi, *n* = 3; **p < 0.01; ***p < 0.001). **(C)** Sections from the mouse intestines were stained with H&E. The infection of the *S.* Typhi-infected *Fut8*^+/–^ mice was found more severe compared to the *S.* Typhi-infected *Fut8*^+/+^ mice. And the infection was expressed in the disruption and swelling of intestinal villus, cell infiltration and thickening of muscular layer.

To investigate the role of core fucosylation during *Salmonella* infection, we infected mice with the *S.* Typhi strain CMCC (B) 50071 CICC 10871 by oral gavage for 7 days (Figure 2A). *S.* Typhi infected *Fut8*^+/–^ mice gained a significant weight loss compared to uninfected *Fut8*^+/–^ mice (*p* < 0.01) and *Fut8*^+/+^ mice (*p* < 0.001) (Figure 2B). To assess the susceptibility of *Fut8*^+/+^ and *Fut8*^+/–^ mice, we stained the small intestine and colon sections of mice of different experimental groups, such as *Fut8*^+/+^, *Fut8*^+/+^ + *S.* Typhi, *Fut8*^+/–^ and *Fut8*^+/–^ + *S.* Typhi groups. The inflammation which was expressed in the disruption and swelling of intestinal villus, cell infiltration and thickening of muscular layer were observed in the intestinal tissues of the *S.* Typhi-infected *Fut8*^+/–^ mice, which was found more severe compared to the *S.* Typhi-infected *Fut8*^+/+^ mice (Figure 2C and Supplementary Figure S2), suggested that low core fucosylation in *Fut8*^+/–^ mice increased the susceptibility to *S.* Typhi infection.

### *S.* Typhi Infection Upregulated the Core Fucosylation Level of IECs in Mice

**FIGURE 3:**
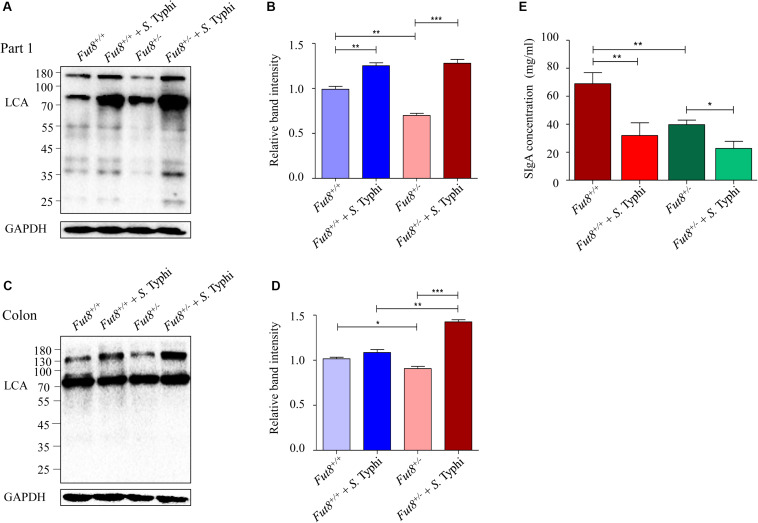
*S.* Typhi infection up-regulated the core fucosylation level of IECs in mice. **(A)** The core fucosylation was up-regulated in intestinal mucosa in the part 1 of small intestine after infected with *S.* Typhi. The core fucosylation levels of intestine tissues detected by means of LCA blotting. **(B)** Expression levels of the core fucosylation in the part 1 of small intestine is assessed by relative band intensity of LCA. Data are shown as mean values ± SEM (*Fut8*^+/+^, *n* = 3; *Fut8*^+/+^ + *S.* Typhi, *n* = 3; *Fut8*^+/–^, *n* = 3; *Fut8*^+/–^ + *S.* Typhi, *n* = 3; ns, not significant; *p < 0.05, **p < 0.01, ***p < 0.001). **(C)** The core fucosylation was up-regulated in intestinal mucosa of colon after *S.* Typhi infection. **(D)** Expression levels of the core fucosylation in colon was assessed by relative band intensity of LCA. Data are shown as mean values ± SEM (*Fut8*^+/+^, *n* = 3; *Fut8*^+/+^ + *S.* Typhi, *n* = 3; *Fut8*^+/–^, *n* = 3; *Fut8*^+/–^ + *S.* Typhi, *n* = 3; ns, not significant; *p < 0.05, **p < 0.01, ***p < 0.001). **(E)** The concentration of sIgA was reduced after *S.* Typhi infection in both *Fut8*^+/+^ and *Fut8*^+/–^ mice, which was measured by sIgA ELISA kit. The concentration of sIgA was found essentially lower in *Fut8*^+/–^ mice. Data are shown as mean values ± SEM (*Fut8*^+/+^, *n* = 3; *Fut8*^+/+^ + *S.* Typhi, *n* = 3; *Fut8*^+/–^, *n* = 3; *Fut8*^+/–^ + *S.* Typhi, *n* = 3; ns, not significant; *p < 0.05, **p < 0.01).

To further investigate the impact of *S.* Typhi infection on IECs core fucosylation, we next examined the levels of core fucosylation in intestinal mucosa of the infected mice. The core fucosylation of *Fut8*^+/–^ mice intestinal mucosa was found significantly lower than that of the *Fut8*^+/+^ mice, especially in the part 1 of small intestine (*p* = 0.002, Figures 3A,B) and colon (*p* = 0.0234, Figures 3C,D). After infection, the core fucosylation of the part 1 of small intestine and colon of *Fut8*^+/+^ mice showed an up-regulated pattern, significantly up-regulated in (part 1 of small intestine, *p* = 0.0047, Figures 3A,B), but without statistic significance in colon (*p* = 0.1357, Figures 3C,D). While the core fucosylation of *Fut8*^+/–^ mice was significantly up-regulated in the part 1 of small intestine (*p* = 0.0003) and colon (*p* = 0.0001). Overall, our data showed that *S.* Typhi infection significantly up-regulated the IECs core fucosylation in mice. In addition, we also analyzed the concentration of sIgA in intestinal content. The concentration of sIgA was obviously down-regulated after *S.* Typhi infection in both *Fut8*^+/+^ (*p* = 0.098) and *Fut8*^+/–^ mice (*p* = 0.0172) (Figure 3E), while it was essentially lower in *Fut8*^+/–^ mice (*p* = 0.0035).

### *S.* Typhi Infection Induced Changes of Gut Microbiota in *Fut8*^+/+^ and *Fut8*^+/–^ Mice

**FIGURE 4:**
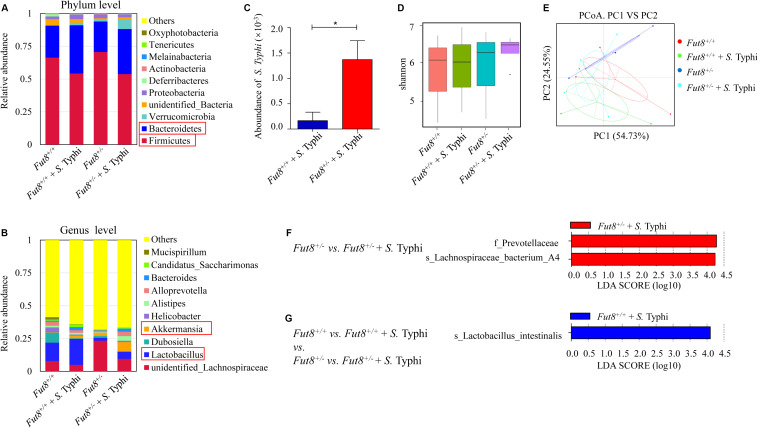
*S.* Typhi infection induced changes of gut microbiota in *Fut8*^+/+^ and *Fut8*^+/–^ mice. **(A)** The relative abundances of the major fecal bacterial phyla in mice. **(B)** The relative abundances of fecal bacterial genera, *Lactobacillus* and *Akkermansia*, were found increased post infection. **(C)** *S.* Typhi was found more abundant in *Fut8*^+/–^ + *S.* Typhi compared with *Fut8*^+/+^ + *S.* Typhi. Data are shown as mean values ± SEM (*Fut8*^+/+^ + *S.* Typhi, *n* = 3; *Fut8*^+/–^ + *S.* Typhi, *n* = 3; *p < 0.05). **(D)** The alpha diversity indicated by Shannon index in each group. **(E)** The beta diversity of fecal microbiota analyzed by principal-coordinate analysis (PCoA). **(F,G)** The bacterial groups that showed significant differences between each group analyzed by the LEfSe method (linear discriminant analysis effect size).

To assess the interaction between IECs core fucosylation and gut microbiota, we investigated the gut microbiota of *Fut8*^+/+^ and *Fut8*^+/–^ mice before and after infection. The relative abundance of the major bacterial phyla in mice belonged to the four experimental groups (*Fut8*^+/+^, *Fut8*^+/+^ + *S.* Typhi, *Fut8*^+/–^, and *Fut8*^+/–^ + *S.* Typhi) were shown in Figure 4A. The major bacterial phyla in gut of mice are *Firmicutes* and *Bacteroides*, they occupied more than 60% of the total bacteria in both *Fut8*^+/+^ and *Fut8*^+/–^ mice, with the abundance of *Firmicutes* obviously higher than that of *Bacteroides*. Interestingly, post infection, the abundance of *Firmicutes* was found decreased in *Fut8*^+/+^ (66.37% vs. 54.28% before and post infection) and *Fut8*^+/–^ mice (70.68% vs. 53.78%), in contrast to the elevation of *Bacteroides* abundance in *Fut8*^+/+^ (24.37% vs. 36.75%) and *Fut8*^+/–^ mice (23.23% vs. 34.38%) (Supplementary Figures S3A,B). Notably, at genus level (Figure 4B), an obvious increase of *Lactobacillus* was detected in both *Fut8*^+/+^ (13.99% vs. 19.76%) and *Fut8*^+/–^ mice (2.17% vs. 5.46%) post *S.* Typhi infection, which was accompanied with an increase of *Akkermansia* in these mice (0. 3% vs. 1.98% in *Fut8*^+/+^ mice; 1.74% *vs.* 7.25% in *Fut8*^+/–^ mice) (Supplementary Figures S3C,D). Also, we detected more colonization of *S.* Typhi in *Fut8*^+/–^ mice post infection when compared with *Fut8*^+/+^ + *S.* Typhi (*p* = 0.0417, Figure 4C). Overall, although the alpha diversity indicated by Shannon index did not reveal a significant difference among the four groups (Figure 4D), a principal-coordinate analysis (PCoA) revealed a significant clustering pattern among them (Figure 4E), after infection the microbiota patterns of *Fut8*^+/+^ and *Fut8*^+/–^ mice shift toward the same direction. All the information about alpha and beta diversity analyses were shown in Supplementary Figures S4, S5. The bacterial groups that showed significant differences between each group were then analyzed by the LEfSe (linear discriminant analysis effect size) method (Figures 4F,G). The family of *Prevotellaceae* and species of *Lachnospiraceae family* A4 were found significantly more abundant in *Fut8*^+/–^ + *S.* Typhi group compared with *Fut8*^+/–^ group (Figure 4F). In addition, comparisons between all the four groups revealed that *Lactobacillus intestinalis* was significantly more abundant in *Fut8*^+/+^ + *S.* Typhi group (Figure 4G), which suggested an important role of *Lactobacillus* in gut of mice during *S.* Typhi infection.

### Wnt Signaling Pathway Along With Core Fucosylation Is Activated After *S.* Typhi Infection

**FIGURE 5:**
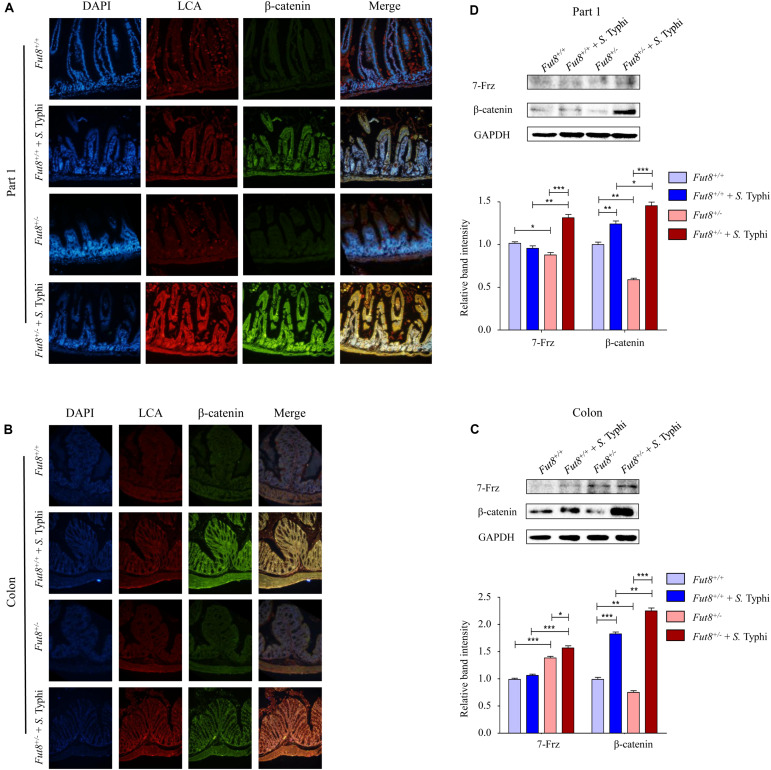
Wnt signaling pathway and core fucosylation were activated in mice after *S.* Typhi infection. **(A)** The core fucosylation and β-catenin expression and localization in mucosa of part 1 of mouse intestine. **(B)** The core fucosylation and β-catenin expression and localization in mucosa of mouse colon. **(C)** Expression levels of 7-Frz and β-catenin in the part 1 of small intestine assessed by Western blotting in intestinal tissues. Data are shown as mean values ± SEM (*Fut8*^+/+^, *n* = 3; *Fut8*^+/+^ + *S.* Typhi, *n* = 3; *Fut8*^+/–^, *n* = 3; *Fut8*^+/–^ + *S.* Typhi, *n* = 3; ns, not significant; *p < 0.05, **p < 0.01, ***p < 0.001). 7-Frz and β-catenin were found up-regulated in intestinal tissues of the part 1 of small intestine after infected with *S.* Typhi. **(D)** Expression levels of 7-Frz and β-catenin in colon. Data are shown as mean values ± SEM (*Fut8*^+/+^, *n* = 3; *Fut8*^+/+^ + *S.* Typhi, *n* = 3; *Fut8*^+/–^, *n* = 3; *Fut8*^+/–^ + *S.* Typhi, *n* = 3; ns, not significant; *p < 0.05, **p < 0.01, ***p < 0.001). 7-Frz and β-catenin were up-regulated in colon after infected with *S.* Typhi.

Next, we assessed the expression of core fucosylation and Wnt signaling pathway of *Fut8*^+/+^ and *Fut8*^+/–^ mice before and after infection in the part 1 and part 4 of small intestine and colon by immunofluorescence and western blot. Frizzled 7 (7-Frz) and β-catenin are the representative protein molecules of Wnt signaling pathway. The immunofluorescence indicated the expression and localization in mucosa of core fucosylation and β-catenin of four groups in part 1 (Figure 5A) and part 4 (Supplementary Figure S1) of small intestine and colon (Figure 5B). Our data showed that core fucosylation and β-catenin were both up-regulated post infection of *S.* Typhi no matter in *Fut8*^+/+^ mice or in *Fut8*^+/–^ mice (Figures 5A,B). 7-Frz was found significantly up-regulated in part 1 of small intestine (Figure 5C, *p* = 0.0012) and colon (Figure 5D, *p* = 0.0189) in *Fut8*^+/–^ mice after *S.* Typhi infection. In addition, β-catenin was also up-regulated in part 1 of small intestine of *Fut8*^+/+^ (*p* = 0.0063) and *Fut8*^+/–^ mice (*p* < 0.0001, Figure 5C), and colon of *Fut8*^+/+^ (*p* < 0.0001) and *Fut8*^+/–^ mice (*p* < 0.0001) (Figure 5D) after *S.* Typhi infection. Overall, our data showed that Wnt signaling pathway and core fucosylation were activated simultaneously after *S.* Typhi infection.

### Wnt Signaling Pathway Mediates the Up-Regulation of Caco-2 Cell Core Fucosylation Stimulated by *S.* Typhi *in vitro*

**FIGURE 6:**
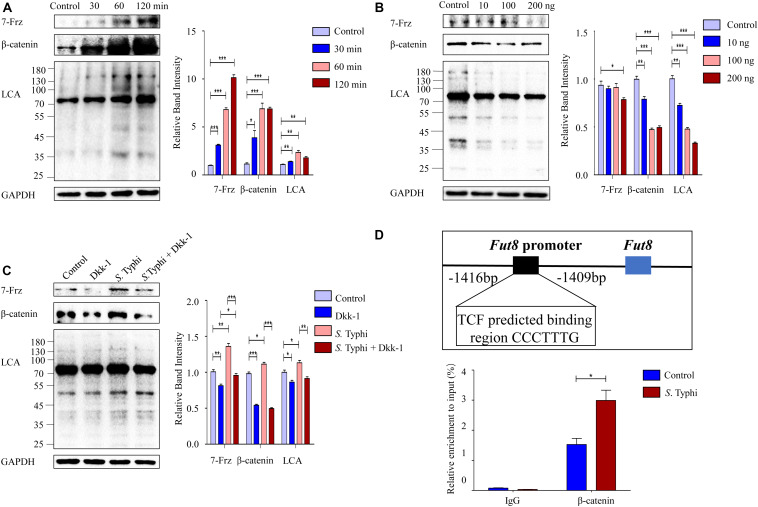
Wnt signaling pathway mediates the core fucosylation of Caco-2 by *S.* Typhi infection *in vitro*. **(A)** The expression of 7-Frz, β-catenin and core fucosylation of Caco-2 cells were up-regulated post infection. Caco-2 cells were incubated with *S.* Typhi for 30 min, washed, and incubated in fresh DMEM with 10% fetal bovine serum for 30, 60, or 120 min. Control group indicates without bacterial treatment. Data are shown as mean values ± SEM (Control, *n* = 3; 30 min, *n* = 3; 60 min, *n* = 3; 120 min, *n* = 3; ns, not significant; *p < 0.05, **p < 0.01, ***p < 0.001). **(B)** The expression of 7-Frz and β-catenin of Caco-2 cells was up-regulated after application of Dkk-1. The cells were divided into control group (group Control) and experimental group (group 10, 100, and 200 ng) according to the completely random method. The dose of Dkk-1 for experimental group: 10, 100, and 200 ng. Data are shown as mean values ± SEM (Control, *n* = 3; 10 ng, *n* = 3; 100 ng, *n* = 3; 200 ng, *n* = 3; ns, not significant; *p < 0.05, **p < 0.01, ***p < 0.001). **(C)** Caco-2 cells were classified into Control group (cells without any treatment), Dkk group (cells were treated by 2 × 10^4^ ng/mL, 10 μl Dkk-1 for 48 h), *S.* Typhi group (cell were incubated with *S.* Typhi strain for 30 min, and incubated with medium for 60 min), *S.* Typhi + Dkk group (cell were incubated with *S.* Typhi strain for 60 min, and incubated with medium for 60 min then treated by 2 × 10^4^ ng/mL, 10 μl Dkk-1 for 48 h). Data are shown as mean values ± SEM (Control, *n* = 3; Dkk-1, *n* = 3; *S.* Typhi, *n* = 3; *S.* Typhi + Dkk-1, *n* = 3; ns, not significant; *p < 0.05, **p < 0.01, ***p < 0.001). **(D)** The interaction between β-catenin/TCF complexes and *Fut8* promoter was analyzed by ChIP analysis. TCF can directly bound to the *Fut8* promoter, and *S.* Typhi infection increased the inputs. Caco-2 cells were incubated with *S.* Typhi for 30 min, washed, and incubated in fresh DMEM with 10% fetal bovine serum for 60 min. Control group indicates without bacterial treatment. Normalized inputs of chromatin DNA from Caco-2 cells were pulled down with anti-β-catenin or negative IgG antibodies. Data are shown as mean values ± SEM (Control, *n* = 3; *S.* Typhi, *n* = 3; *p < 0.05).

To determine whether core fucosylation is regulated through Wnt signaling pathway, we infected Caco-2 cells with *S.* Typhi and found that the expression levels of 7-Frz and β-catenin were significantly increased post *S.* Typhi infection with a time-dependent pattern (Figure 6A). Consistently, we also found up-regulation of the core fucosylation levels of Caco-2, suggesting that the up-regulation of the core fucosylation may correlate with Wnt signaling pathway. To further determine this correlation, we investigated the effect of Wnt signaling antagonist, Dickkopf-1 (Dkk-1), which functions by binding to the LRP5/LRP6 helper receptor and inhibiting canonical Wnt signaling pathway (Nakamura et al., 2003). The expression of 7-Frz and β-catenin were significantly decreased in Caco-2 after the application of Dkk-1 with a concentration-dependent pattern (Figure 6B). Consistently, we also found a similar down-regulation of the core fucosylation levels in Caco-2. Next, we analyzed the effect of *S.* Typhi infection after the application of Dkk-1. As shown in Figure 6C, Caco-2 cells were divided into four group (group Control, Dkk, *S.* Typhi, and *S.* Typhi + Dkk-1), we found that *S.* Typhi no longer increase the expression of 7-Frz, β-catenin and the expression of the core fucosylation with the application of Dkk-1. Therefore, we sought to clarify whether β-catenin directly regulates Fut8 expression or not. There are the promoter regions of *Fut8* gene upstream of the transcription initiation site. To further validate *Fut8* is one of the downstream targets of the Wnt/β-catenin signaling pathway, we predicted a putative β-catenin-T cell factor (TCF)-binding site (5′-CCCTTTG-3′) on *Fut8* promoter regions, and infected Caco-2 cells with *S.* Typhi. In the CHIP experiments, the β-catenin specifically bound to the *Fut8* promoter region (−1416 bp to −1409 bp) (*p* = 0.021) (Figure 6D). Collectively, these results indicated that Wnt signaling pathway mediated the up-regulation of core fucosylation stimulated by *S.* Typhi.

## Discussion

Core fucosylation is a common type of post-translational protein modification, and it is involved in a variety of physiological and pathological processes, such as Crohn’s disease and colorectal cancer (Muinelo-Romay et al., 2008; Franke et al., 2010; McGovern et al., 2010). Understanding the mechanisms of regulation of Fut8 gene expression and core fucosylation, one of the major glycosylation events in IECs, is therefore of great important. Our results proved that core fucosylation is ubiquitous in IECs, and core fucosylation is closely related to abundance of *Lactobacillus* spp. It has been reported that the expression of epithelial fucosylation is higher in the ileum (part 4 of intestine), which harbors more abundant gut microbes than in the duodenum (part 1) (Barker et al., 2007; Goto et al., 2014). Our results also proved that the core fucosylation of part 4 is significantly higher than other parts of intestine. Our recent study also revealed that *Lactobacillus* and *Bifidobacterium* specifically organisms synthesize α-L-fucosidase that cleave core fucose residue, and once inside the bacteria, the fucose was catabolized. Therefore, the core fucosylated *N*-glycans can facilitate the growth of *Lactobacillus* in gut of infants (Li et al., 2019).

Given that the commensal bacteria of the gastrointestinal tract play an important role in protecting the host from pathogenic bacteria infection to maintain intestinal homeostasis (Mueller and Macpherson, 2006; Backhed et al., 2012). The imbalance of microbiota caused by pathogen infection is considered to be closely related to inflammation, obesity, fucosylation, sialylation and other metabolic factors (Boulange et al., 2016; Zhou et al., 2020). It has been reported that *Salmonella* infection enhanced the levels of proinflammatory cytokines IL-1β, IL-8, and LITNF. And the imbalance of microbiota can increase susceptibility to pathogenic infection (Brown et al., 2013; Moreira et al., 2017). For instance, antibiotic treatment alters the microbial community and predisposes the host to *Salmonella* infection (Sekirov et al., 2008). Our study showed that the deficiency of core fucosylation affected intestinal microbiota in mouse, and *Fut8*^+/–^ mice was more susceptive to *S.* Typhi infection when compared with *Fut8*^+/+^ mice, this suggested an interaction between IECs core fucosylation and gut microbiota. Fut2-mediated epithelial fucosylation (α1,2 fucosylation) is initiated by direct interaction between commensals and IECs (Goto et al., 2014). Commensal bacteria, for example the *Bacteroides* spp., can use focuses as components of the bacterial outer membrane, or use them as a nutrient (Leis et al., 1997; Coyne et al., 2005). Moreover, some glycans are able to resist pathogens and shape commensal bacterial structure (Hooper et al., 1999). As we found that the deficient of core fucosylation, *Lactobacillus* is decreased in *Fut8*^+/–^ mice, therefore, the epithelial core fucose reacts as a mediator between the host and commensal microbiota. Based on these data, it can be concluded that core fucosylation can promote the colonization of commensal bacteria like *Bacteroides fragilis*, *Lactobacillus*, and *Akkermansia*. When assessing the microbiota community structure of *Fut8*^+/+^ and *Fut8*^+/–^ mice, we also found that the abundance of *Lactobacillus* and *Akkermansia* was increased in mice after *S.* Typhi infection. It has been reported that lactic acid and hydrogen peroxide, the main metabolites of *Lactobacillus*, act in a cooperative way to resist enteric and vaginosis-associated pathogens (Servin, 2004; Fayol-Messaoudi et al., 2007). *Akkermansia* not only has the capacity to degrade mucins, but also to stimulate mucin synthesis to clear environmental antigens, acting as innate host defense and protection against to infections (McGuckin et al., 2011; Shin et al., 2014). Furthermore, the mucin degradation activity of *Akkermansia* leads to the production of short-chain fatty acids (SCFAs), which can form an acidic environment to inhibit the growth of *S.* Typhi (Derrien et al., 2004; Shin et al., 2014). It can be concluded that the increase of *Lactobacillus* and *Akkermansia* is antagonistic to *S.* Typhi infection. Furthermore, there are some studies showed that bacterial flagellin of *S.* Typhi can induce Th17 cells which promotes inflammation, and *Lactobacillus* have the potential to induce Treg cells which promotes immune tolerance (Hansson and Johansson, 2010; Round and Mazmanian, 2010; Round et al., 2011; Goto and Kiyono, 2012). Moreover, intestinal APCs such as dendritic cells (DCs) and macrophages (MPs) play a pivotal role in suppressing microbiota-induced inflammation and mediating mucosal tolerance (Staal et al., 2008; Maloy and Powrie, 2011; Rescigno, 2011; Swafford et al., 2018). It has also been reported that cytokines interleukin-22 and lymphotoxin producing type 3 innate lymphoid cells (ILC3) regulate epithelial fucosylation on IECs to inhibit *S.* Typhimurium infection (Goto et al., 2014). Secretory IgA (sIgA) functioned by limiting pathogen access to the mucosal surface and protecting immunity toward *Salmonella* infection (Endt et al., 2010). In addition, the damaged muscular layer was observed in the intestinal tissues of the *S.* Typhi-infected *Fut8*^+/–^ mice, and the concentration of sIgA is obviously reduced after *S.* Typhi infection by a damage of mucosal immune function (Tokuhara et al., 2010). Our data thus suggest that *S.* Typhi induced up-regulation of IECs core fucosylation, which promoted the abundance of core-fucose utilizing bacteria such as *Lactobacillus* and *Akkermansia*, to resist the invasion of *S.* Typhi, and this interaction contributed to the improvement of the intestinal barrier function and immune responses subsequently.

It has been reported that *Salmonella* infection causes the activation of canonical Wnt signaling pathway which plays an important role in embryonic development and tumorigenesis, such as intestinal renewal, inflammation and colorectal cancer (Liu et al., 2010; Wang et al., 2018). Wnt signaling pathway was also significantly activated after *S.* enterica serovar Enteritidis infection, characterized by GSK-3β and β-catenin phosphorylated (Kogut and Arsenault, 2015). The most relevant limitation of this study is the use of pharmacological drugs as unique tool to demonstrate the mechanism underlying the fucosylation modification on IECs, although we used Dickkopf-1 (Dkk-1), a wnt signaling antagonist, which functions by binding to the LRP5/LRP6 helper receptor and inhibiting canonical Wnt signaling pathway (Nakamura et al., 2003). Our study showed that the Wnt signaling pathway is activated post *S.* Typhi infection, and the application of Wnt signaling pathway antagonist Dkk-1suppressed IECs core fucosylation. Our study also demonstrated that β-catenin can directly bound to the *Fut8* promoter regions of *Fut8* gene. These data indicated that the *S.* Typhi-induced activation of Wnt signaling pathway leads to the accumulation of β-catenin and enhances *Fut8* transcription, contributes to the rapid elevation of IECs core fucosylation.

**FIGURE 7:**
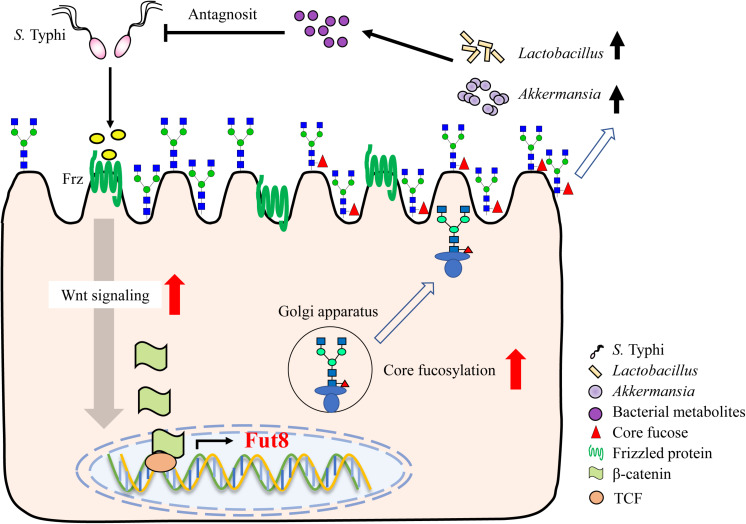
Core fucosylation of IECs protects against *S.* Typhi infection via up-regulating the biological antagonism of intestinal microbiota.

An immense structural diversity of IECs glycans contribute to the intestinal microbiota formation. Thus, their importance for both host and intestinal bacteria in long-term commensal interactions is beginning to be appreciated. A key finding of our study is the core fucosylation on IECs is closely associated with the intestinal microbiota formation and invasion of *S.* Typhi. During *S.* Typhi infection, the Wnt signaling pathway, represented by the expression of Frizzled protein and β-catenin, is rapidly up-regulated, resulted in elevation of epithelial core fucosylation. The abundant core fucose on the surface of IECs facilitates the growth of fucose-utilizing commensal bacteria such as *Lactobacillus* and *Akkermansia*, who producing lactic acid and other metabolites to resist the invasion of *S.* Typhi (Figure 7).

## Data Availability Statement

The raw data supporting the conclusions of this article will be made available by the authors, without undue reservation, to any qualified researcher.

## Ethics Statement

All animal work was approved by the Ethics Committee (approval No. AEE17013) at the Dalian Medical University, Dalian, China.

## Author Contributions

WL, ML, and SH designed the research. SH, QF, YB, HF, and JZ performed the experiments. SH analyzed the experimental data and wrote the manuscript. ML, WL, TF, and JG corrected the manuscript. All authors reviewed the results and approved the final version of the manuscript.

## Conflict of Interest

The authors declare that the research was conducted in the absence of any commercial or financial relationships that could be construed as a potential conflict of interest.

## Supplementary Material

FIGURE S1 Wnt signaling pathway along with core fucosylation is activated after *S. typhi* infection in part 4 of small intestine. **(A)** The core fucosylation and β-catenin expression and localization in mucosa of part 4 of intestine.

FIGURE S2 The pathology score of small intestine and colon. **(A)** The pathology score of part 1 of small intestine. **(B)** The pathology score of part 4 of small intestine. **(C)** The pathology score of colon. Data are shown as mean values ± SEM (*Fut8*^+/+^, *n* = 3; *Fut8*^+/+^ + *S.* Typhi, *n* = 3; *Fut8*^+/–^, *n* = 3; *Fut8*^+/–^ + *S.* Typhi, *n* = 3; ns, not significant; ^∗^*p* < 0.05, ^∗∗^*p* < 0.01, ^∗∗∗^*p* < 0.001).

FIGURE S3 **(A)** Relative abundance of *Firmicutes*. **(B)** Relative abundance of *Bacteroidetes*. **(C)** Relative abundance of *Lactobacillus*. **(D)** Relative abundance of *Akkermansia*. Data are shown as mean values ± SEM (*Fut8*^+/+^, *n* = 3; *Fut8*^+/+^ + *S.* Typhi, *n* = 3; *Fut8*^+/–^, *n* = 3; *Fut8*^+/–^ + *S.* Typhi, *n* = 3; ns, not significant).

FIGURE S4 The alpha diversity of fecal microbiota. **(A)** The alpha diversity indicated by ACE index in each group. **(B)** The alpha diversity indicated by chao 1 index in each group. **(C)** The alpha diversity indicated by goods coverage index in each group. **(D)** The alpha diversity indicated by observed species index in each group. **(E)** The alpha diversity indicated by PD whole tree index in each group. **(F)** The alpha diversity indicated by simpson index in each group.

FIGURE S5 The beta diversity of fecal microbiota. **(A)** The beta diversity indicated by weighted unifrac index in each group. **(B)** The beta diversity of fecal microbiota analyzed by NMDS. **(C)** The beta diversity of fecal microbiota analyzed by PCA. **(D)** The beta diversity of fecal microbiota analyzed by heatmap.
