# Supplementary figures and images for: Core Fucosylation of Intestinal Epithelial Cells Protects Against Salmonella Typhi Infection via Up-Regulating the Biological Antagonism of Intestinal Microbiota
Source: Front Microbiol. 2020 May 27;11:1097. doi: 10.3389/fmicb.2020.01097 (PMC7266941; doi:10.3389/fmicb.2020.01097)

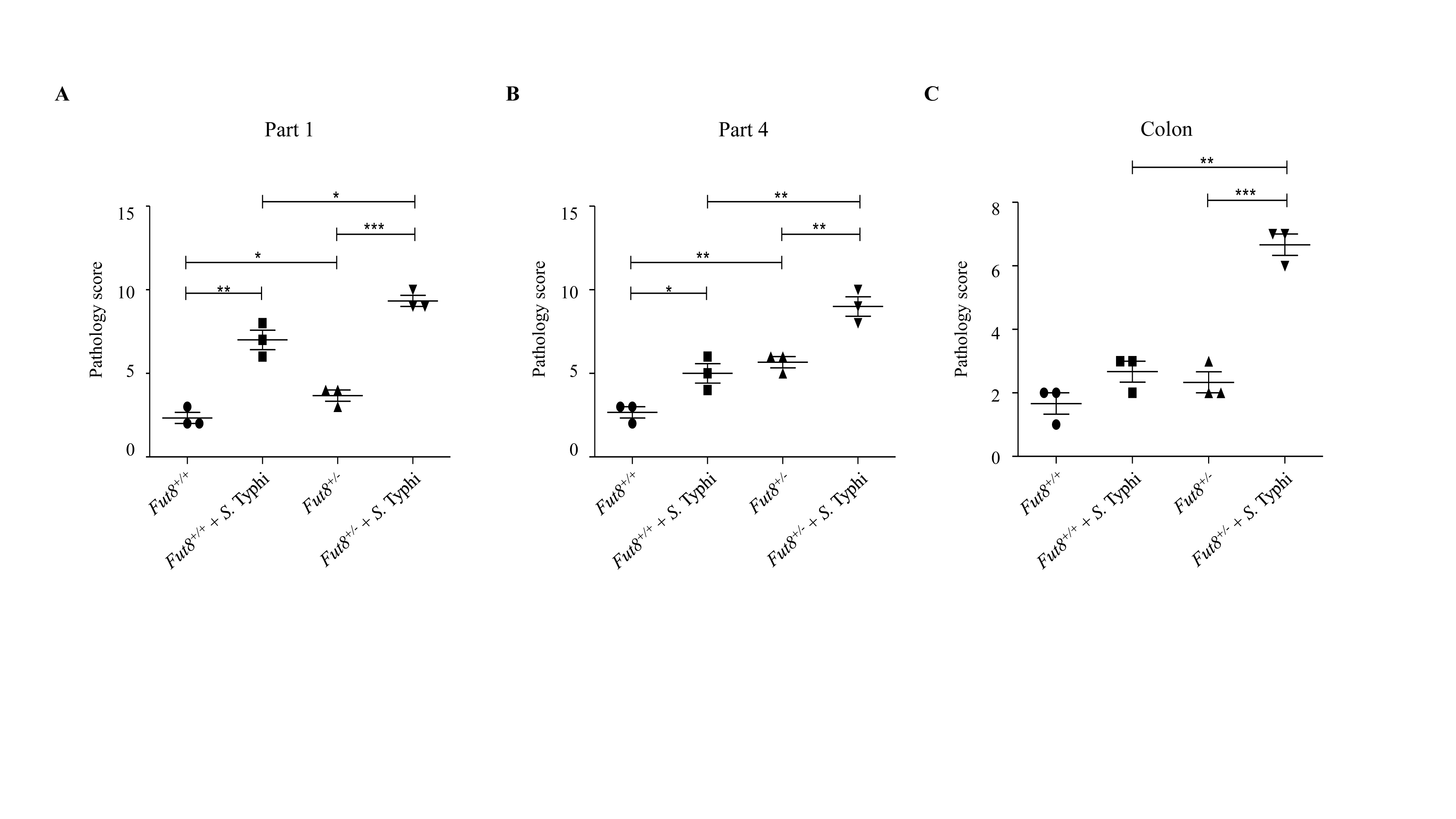

Supplement: FIGURE S2 — The pathology score of small intestine and colon. (A) The pathology score of part 1 of small intestine. (B) The pathology score of part 4 of small intestine. (C) The pathology score of colon. Data are shown as mean values ± SEM (Fut8+/+, n = 3; Fut8+/+ + S. Typhi, n = 3; Fut8+/–, n = 3; Fut8+/– + S. Typhi, n = 3; ns, not significant; ∗p < 0.05, ∗∗p < 0.01, ∗∗∗p < 0.001). [file Image_2.TIF]

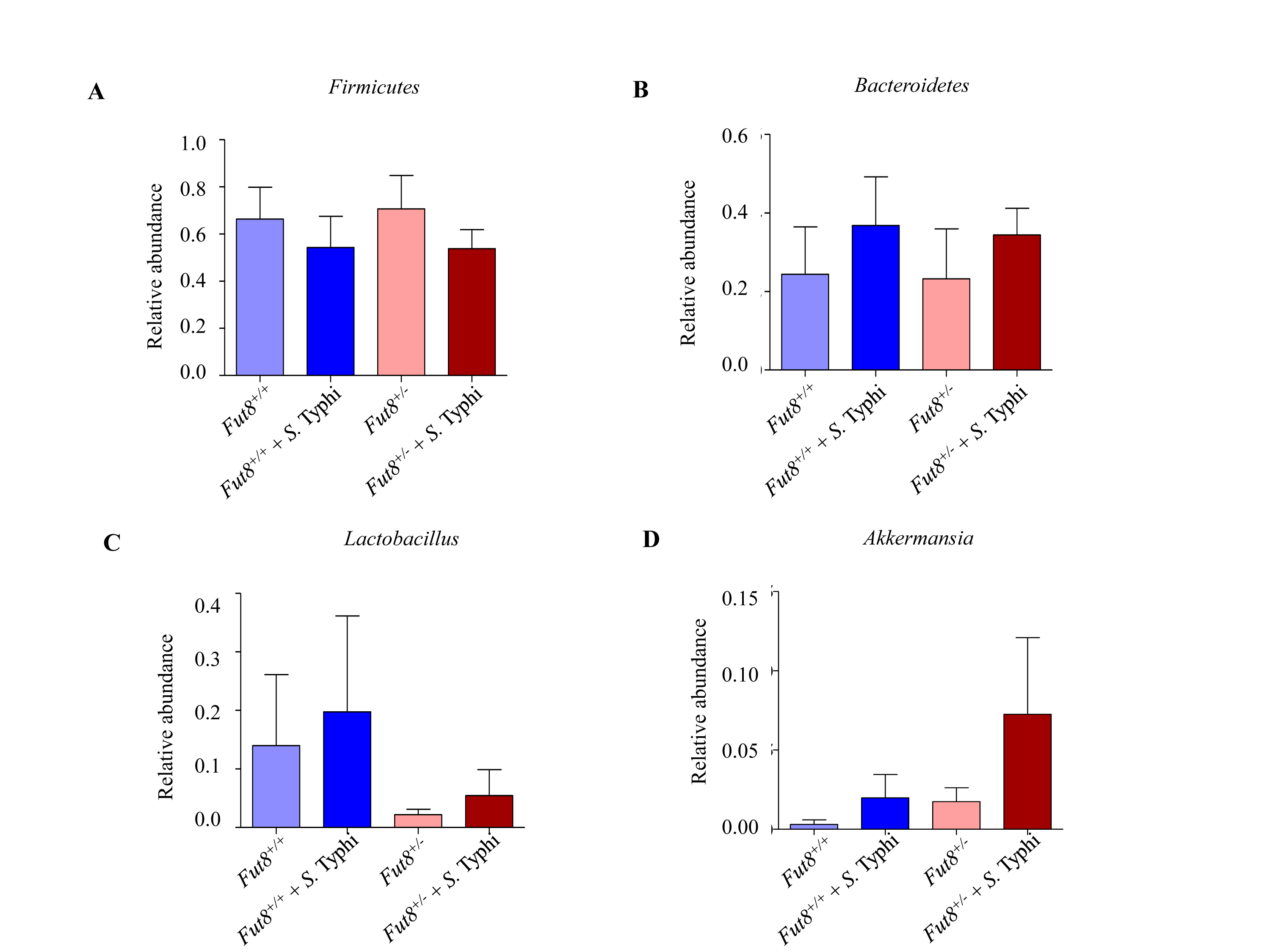

Supplement: FIGURE S3 — (A) Relative abundance of Firmicutes. (B) Relative abundance of Bacteroidetes. (C) Relative abundance of Lactobacillus. (D) Relative abundance of Akkermansia. Data are shown as mean values ± SEM (Fut8+/+, n = 3; Fut8+/+ + S. Typhi, n = 3; Fut8+/–, n = 3; Fut8+/– + S. Typhi, n = 3; ns, not significant). [file Image_3.TIF]

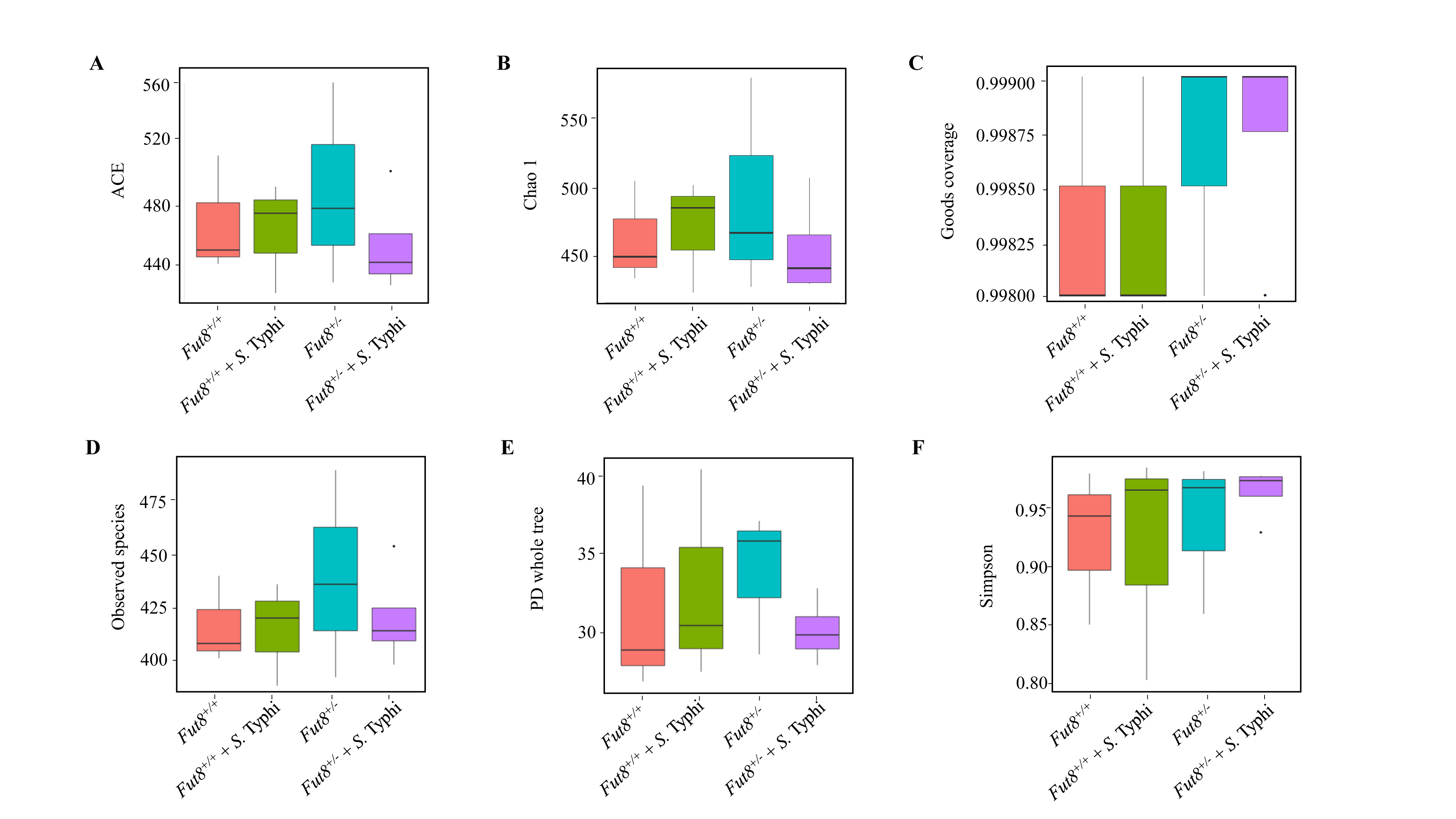

Supplement: FIGURE S4 — The alpha diversity of fecal microbiota. (A) The alpha diversity indicated by ACE index in each group. (B) The alpha diversity indicated by chao 1 index in each group. (C) The alpha diversity indicated by goods coverage index in each group. (D) The alpha diversity indicated by observed species index in each group. (E) The alpha diversity indicated by PD whole tree index in each group. (F) The alpha diversity indicated by simpson index in each group. [file Image_4.TIF]

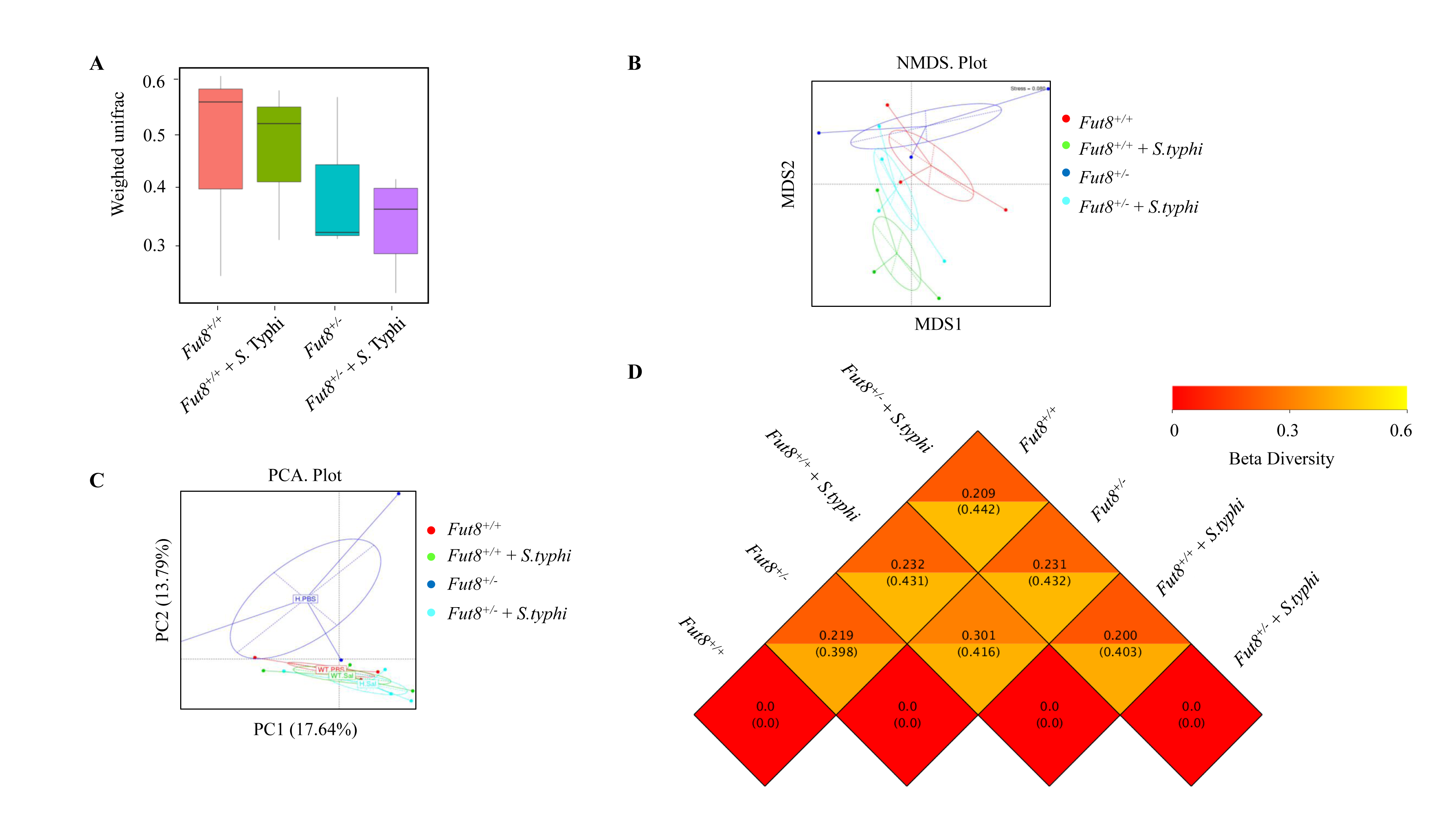

Supplement: FIGURE S5 — The beta diversity of fecal microbiota. (A) The beta diversity indicated by weighted unifrac index in each group. (B) The beta diversity of fecal microbiota analyzed by NMDS. (C) The beta diversity of fecal microbiota analyzed by PCA. (D) The beta diversity of fecal microbiota analyzed by heatmap. [file Image_5.TIF]
